# The financial impact of neonatal sepsis on the Brazilian Unified Health System

**DOI:** 10.1016/j.clinsp.2023.100277

**Published:** 2023-08-28

**Authors:** Mariana Ferreira Carvalho Westerstahl de Abreu, Joice Fabíola Meneguel Ogata, Marcelo Cunio Machado Fonseca, Dayan Sansone, Ruth Guinsburg

**Affiliations:** a Division of Neonatal Medicine, Department of Pediatrics, Escola Paulista de Medicina, Universidade Federal de São Paulo, São Paulo, SP, Brazil; b Department of Gynecology, Escola Paulista de Medicina, Universidade Federal de São Paulo, São Paulo, SP, Brazil; c Health Technology Assessment Center, Department of Gynecology, Escola Paulista de Medicina, Universidade Federal de São Paulo, São Paulo, SP, Brazil; d Department of Pediatrics, Escola Paulista de Medicina, Universidade Federal de São Paulo, São Paulo, SP, Brazil

**Keywords:** Neonatal sepsis, Costs, Delivery of health care, Health information systems

## Abstract

**Objective:**

To evaluate the hospital cost of newborn infants diagnosed with sepsis from the perspective of the Brazilian Public Health System over 11 years.

**Method:**

Cross-sectional study that analyzed secondary data from the databases of the Hospital Information System of the Brazilian Public Health System. Infants hospitalized between 0‒29 days after birth with a diagnosis of sepsis from 2008 to 2018 were included. The diagnosis used in the study was the one that the hospital considered the main diagnosis at admission. Costs were analyzed in US dollars and reflected the amount paid by the Brazilian Public Health System to the hospitals for the informed diagnosis upon admission. The costs were evaluated as the total per admission, and they were compared among Brazilian geographic regions, among etiologic agents, and between neonates with the diagnosis of sepsis that survived or died.

**Results:**

From 2008 to 2018, 47,554 newborns were hospitalized with sepsis (148.04 cases per 100,000 live births), with an average cost of US$ 3345.59 per hospitalization, ranging from US$ 2970.60 in the North region to US$ 4305.03 in the Midwest. Among sepsis with identified agents, the highest mean cost was related to Gram-negative agents, and the lowest to *Streptococcus agalactiae* sepsis. Patients with sepsis who died had a higher cost than the survivors (*t*-test; *p* = 0.046).

**Conclusions:**

The evaluation of costs related to neonatal sepsis in the country during an 11-year period shows the economic impact of morbidity that may be avoided by improving the quality of neonatal care.

## Highlights

• Understanding the costs of neonatal sepsis helps to provide the problem's dimension.
• Mean hospital cost for neonatal sepsis was US$ 3345.59 per patient in 2008‒2018.
• Improvement in quality of care may decrease neonatal sepsis’ economic impact.

## Introduction

Currently, the main causes of neonatal deaths, in the global scenery, are complications of prematurity (35%), intrapartum events (24%) and sepsis (15.6%).^1^ Neonatal infections are associated, in addition to mortality, to long-term impairments in the survivors.^2^ In low and medium-income countries, neonatal deaths in developing countries could be largely avoided with the application of simple and low-cost resources, including the prevention of neonatal sepsis.^1^

In the context of health economics, it is necessary to distinguish financial and economic costs. Oliveira et al.,^1^ in 2014, defined financial costs as the use of real money in resources necessary to carry out an intervention. Economic costs include, in addition to the direct use of money, the value of resources for which no monetary value has actually been spent, in order to provide a more realistic estimate of the financial costs. Knowledge of hospital costs is essential for the planning, management, and administration of health services, whether in the public or private sphere.^3^ Cost analysis encompasses the identification, quantification, and recognition of all resources used in health care.^4^

A broader understanding of costs related to neonatal sepsis may provide the real dimension of the problem, enabling the creation of cost-effective strategies for its prevention, directing public policies and providing better financial management by managers and governors.^5^, ^6^, ^7^ A systematic review of 37 publications on the cost of sepsis for the hospital system in adults concluded that the mean cost of hospitalization for sepsis per patient was US$ 32,421.00, ranging from US$ 20,745.00 to US$ 40,835.00.^8^ In 2018, an analysis of the economic impact of neonatal sepsis in Sub-Saharan Africa concluded that 5.29 to 8.73 million Disability-Adjusted Life Years (DALY) are lost annually due to neonatal sepsis, with an estimated estimated impact ranging from US$ 10 billion to US$ 469 billion per year.^9^ In Brazil, Neira et al. published a multicenter study on the epidemiology and costs of sepsis between 2006‒15, encompassing patients from zero to over 85 years of age; the average cost per patient hospitalized with sepsis was US$ 624.00. When there was a need for intensive care, the average cost per patient increased to US$ 1,798.00. However, there are no specific data for the neonatal group.^6^

In this context, the aim of this study was to explore and describe the economic cost of neonatal sepsis in the studied country, from the perspective of the Unified Health System, verifying whether this cost differs between regions of Brazil, between the different etiological agents and among those newborns who die or survive after sepsis.

## Methods

This is a cross-sectional study, with analysis of secondary data through the evaluation of one of the databases of the Department of Informatics of the Unified Health System, compiled by the Hospital Information System of the Unified Health System (DATASUS, SIH/SUS),^10^ which includes hospitalizations for neonatal sepsis from 2008 to 2018. The study was approved by the Ethics and Research Committee of the Universidade Federal de São Paulo (# 3.127.985).

Access to the database is free on the DATASUS website (www.datasus.gov.br). The available data come from the Hospital Information System of the Unified Health System (SIH/SUS), managed by the Ministry of Health. The hospital units participating in the SUS send information on hospitalizations through the Hospital Admission Authorization (AIH) to the system. This information is processed in DATASUS, generating credits for the services provided and establishing a database with information on hospital admissions carried out in Brazil. Tables were built with the data of interest through a program in Python language especially developed to extract information from the .dbf files provided by DATASUS.

In the database specified above, the authors studied all hospitalized patients aged less than 30 days who, at admission, were reported as having pre-established diagnoses associated with neonatal sepsis, according to the International Classification of Diseases, ICD 10: A021, A267, A327, A40, A401-A403, A408-A415, A418, A419, A427, B377, P360-P365, P368 and P369.^11^ The study period was established due to the standardization of the database consolidation criteria in 2008.

Costs are presented in US dollars, calculated by the amount paid for hospitalization on a given day and year multiplied by the monthly average of the dollar, as reported by the Institute of Applied Economic Research, linked to the Ministry of Economy.^12^ Monetary correction was not performed, since the value did not change during the years of the study.^13^

As it is a descriptive study of the entire Brazilian population under the age of 30 days hospitalized for sepsis in the years 2008 to 2018, there was no sample size calculation. The values transferred by SUS for each hospitalization were described in average, maximum and minimum cost. The non-parametric Kruskal-Wallis test was applied to compare the costs among the regions of the country and among the different agents. For the *post hoc* analysis of the difference in costs between specific regions and between etiologic agents, *pairwise* comparisons were used. The comparison of the average cost of newborns who died and those who survived was performed using Student's *t*-test once normal conditions were met. A significance level of 5% (α = 0.05) was adopted. All tests were performed using SPSS version 26 (IBM Corp, NY, United States). The manuscript followed the “Strengthening the Reporting of Observational Studies in Epidemiology” (STROBE) guidelines.

## Results

Between 2008 and 2018, DATASUS recorded 32,122,645 live births in Brazil. During this period; 47,554 newborns were hospitalized with the main diagnosis of neonatal sepsis. Considering these numbers, the reported frequency of neonatal sepsis was 148.04 cases per 100,000 live births in the Brazilian public system. The distribution of hospitalizations for neonatal sepsis in relation to the number of live births for each year of the study is shown in Table 1.

**Table 1:** Hospitalizations for neonatal sepsis by geographic region during the study period according to year of birth.

Year	LB	Hospitalizations due to sepsis	Hospitalizations due to sepsis by region					% of LB hospitalized due to sepsis
			MW	NE	N	SE	S	
2008	2,934,828	3042	145	405	156	1353	983	0.10
2009	2,881,581	3243	84	406	226	1573	954	0.11
2010	2,861,868	3810	103	445	303	1981	978	0.13
2011	2,913,160	3902	115	575	305	2017	890	0.13
2012	2,905,789	4200	131	774	260	2115	920	0.14
2013	2,904,027	4245	122	938	232	2041	912	0.15
2014	2,979,259	4347	155	1155	173	1964	900	0.15
2015	3,017,668	5006	131	1365	308	2107	1095	0.17
2016	2,857,800	5172	206	1228	379	2292	1067	0.18
2017	2,923,535	5083	177	1237	318	2342	1009	0.17
2018	2,943,130	5504	300	1255	343	2445	1161	0.19
**Total**	**32,122,645**	**47,554**	**1,669**	**9783**	**3003**	**22,230**	**10,869**	**0.15**

MW, Midwest; NE, Northeast; N, North; SE, Southeast; S, South; LB, Live Births.

The values of hospitalizations for neonatal sepsis in the different Brazilian regions are shown in Table 2. There was a difference among regions regarding the cost of hospitalizations for sepsis (Kruskall–Wallis; *p <* 0.001). In the *post hoc* analysis using the *pairwise* method adjusted by Bonferroni correction, the Midwest region had the highest cost of hospitalizations compared to all other regions (*p <* 0.001). There was also a higher cost in the comparisons of the Southeast region with the North, Northeast and South regions (*p <* 0.001) and the South region in relation to the North region (*p* = 0.018).

**Table 2:** Number of SUS hospitalizations due to neonatal sepsis in Brazil between 2008 and 2018 and average, maximum and minimum cost (in US dollars), according to the Unified Health System, according to the regions of the Federation.

	Hospitalizations, n (%)	Mean cost*	Maximum cost	Minimum cost
**North ‒ N**	**3003 (6.3%)**	**2970.60**	**22,568.57**	**163.86**
PA	1443 (3.0%)	3650.04	22,568.57	218.54
RO	584 (1.2%)	1970.46	12,846.74	173.75
TO	429 (0.9%)	2401.02	19,430.15	196.10
AM	401 (0.8%)	3103.12	21,117.55	185.82
AC	88 (0.2%)	1384.91	6223.45	163.86
RR	57 (0.1%)	1868.43	12,573.19	331.15
AP	1 (0.0%)	189.61	189.61	189.61
**Northeast ‒ NE**	**9783 (20.6%)**	**3362.08**	**55,680.52**	**101.95**
CE	2991 (6.3%)	5045.78	55,680.52	179.97
PB	1990 (4.2%)	1746.65	14,980.94	101.95
BA	1645 (3.5%)	3432.59	27,590.32	173.47
PE	843 (1.8%)	3316.69	18,842.16	196.03
AL	758 (1.6%)	2362.08	12,381.32	167.09
RN	625 (1.3%)	2559.79	19,492.48	200.45
MA	606 (1.3%)	2755.42	15,830.06	245.28
SE	182 (0.4%)	2911.39	20,613.85	186.67
PI	143 (0.3%)	2034.24	13,224.13	193.54
**Southeast ‒ SE**	**22,230 (46.7%)**	**3464.80**	**33,785.70**	**120.05**
MG	9755 (20.5%)	3758.44	32,623.95	160.36
SP	8355 (17.6%)	3505.95	32,866.74	120.05
RJ	2335 (4.9%)	2858.35	33,785.70	176.89
ES	1785 (3.8%)	2460.70	32,362.81	214.54
**South ‒ S**	**10,869 (22.9%)**	**3043.20**	**33,067.53**	**150.24**
RS	7757 (16.3%)	2826.45	33,067.53	150.24
PR	2146 (4.5%)	3720.05	33,038.67	173.75
SC	966 (2.0%)	3280.05	23,800.79	226.27
**Midwest ‒ MW**	**1669 (3.5%)**	**4305.03**	**41,242.73**	**214.54**
DF	709 (1.5%)	4554.44	41,242.73	214.54
MT	484 (1.0%)	4659.50	22,517.69	251.31
GO	368 (0.8%)	3757.40	16,210.47	218.41
MS	108 (0.2%)	2945.11	17,184.22	217.38
**Total**	**47,554 (100.0%)**	**3345.59**	**55,680.52**	**101.95**

Mean hospitalization costs differences among regions: Kruskal–Wallis; *p*-value < 0.001. Pairwise pos-hoc comparisons: MW > N; MW > NE; MW > SE; MW > S; SE > N; SE > S (all with *p*-value < 0.001) and S > N (*p*-value = 0.018).

The cost of sepsis to the national public health system due to different etiological agents is described in Table 3. A higher cost of hospitalizations for Gram-negative bacteria was observed, compared to streptococci (*p <* 0.001) and unspecified agents (*p <* 0.001). There was also a higher cost of sepsis by *S. aureus* compared to streptococci (*p <* 0.001) and unspecified agents (< 0.001). There was also a difference in the costs of hospitalizations for anaerobes and *Listeria monocytogenes*, compared to streptococci (*p <* 0.001) and unspecified agents (*p <* 0.001), being higher for the first ones.

**Table 3:** Total number of SUS hospitalizations due to neonatal sepsis in Brazil in the period 2008‒2018 and average, maximum and minimum cost (in US dollars), according to the Unified Health System, according to the different etiological agents.

	Hospitalizations, n (%)	Mean cost	Maximum cost	Minimum cost
Unspecified agents	42,182 (88.8%)	3312.40	55,680.52	101.95
Unspecified staphylococci	1792 (3.8%)	3694.90	28,515.92	163.86
Other staphylococci	1676 (3.5%)	3282.69	29,051.73	347.45
Gram negative	1029 (2.2%)	4435.94	41,242.73	189.61
*Streptococcus agalactiae*	304 (0.6%)	2418.00	26,332.00	181.36
*Stafilococcus aureus*	240 (0.5%)	4243.33	21,127.02	167.09
Streptococcus not specified	225 (0.5%)	2420.51	15,821.33	185.82
Anaerobes	81 (0.2%)	3778.25	12,608.04	325.82
*Listeria monocytogenes*	25 (0.1%)	3223.29	12,637.10	456.14
**Total**	**47,554 (100.0%)**	**3345.59**	**55,680.52**	**101.95**

Mean hospitalization costs differences among different agents: Kruskal-Wallis; *p*-value < 0.001. Pairwise post-hoc comparisons: Gram negative > streptococci; Gram negative > unspecified agents; *Stafilococcus aureus* > streptococci; *Stafilococcus aureus* > unspecified agents; *Listeria* > streptococci; *Listeria* > unspecified agents. All post-hoc tests with *p*-value < 0.001.

Table 4 shows the cost of neonatal hospitalizations for sepsis from the SUS perspective for those infants whose outcome was death or survival, with a higher cost for patients who died (*t*-test; *p* = 0.046).

**Table 4:** Number of SUS hospitalizations for neonatal sepsis in Brazil from 2008‒2018 whose outcome was death or survival and the respective cost (in US dollars) for the Brazilian public system of these hospitalizations, according to the regions of the Federation.

	Hospitalizations, n (%)	Total cost	Mean cost	Maximum cost	Minimum cost
**Deaths**					
Midwest	293	1,146,216.25	3912.00	41,242.73	214.54
Southeast	2484	9,210,861.58	3708.08	32,315.58	160.36
Northeast	1963	6,420,514.85	3270.77	30,430.21	101.95
South	654	2,075,163.64	3173.03	23,710.30	177.55
North	569	1,634,236.81	2872.12	17,164.09	173.75
**Total**	**5,963**	**20,486,993.13**	**3435.69**	**41,242.73**	**101.95**
**Suvirvors**					
Midwest	1376	6,038,875.64	4388.72	30,502.69	251.31
Southeast	19,746	67,811,554.19	3434.19	33,785.70	120.05
Northeast	7820	26,470,703.05	3385.00	55,680.52	126.24
South	10,215	31,001,394.30	3034.89	33,067.53	150.24
North	2434	7,286,471.98	2993.62	22,568.57	163.86
**Total**	**41,591**	**138,608,999.17**	**3332.67**	**55,680.52**	**120.05**

Deaths vs survivors: Student *t*-test; *p*-value = 0.046.

## Discussion and conclusion

Bacterial sepsis is an important condition in the neonatal population, with high morbidity and mortality and economic implications in terms of hospital costs, costs related to childhood morbidity, and socioeconomic insertion of survivors into adulthood.^14^^,^^15^ This study assessed part of the economic impact of neonatal sepsis.

In an attempt to understand the results, attention is drawn, first of all, to the incidence of hospitalizations with neonatal sepsis of 148.04 cases per 100,000 live births, which is lower than the incidence of neonatal sepsis reported in a 2018 meta-analysis, covering high-income countries and middle income: the incidence ranged from 450 to 17,000 cases of neonatal sepsis per 100,000 live births, with large variations depending on the geographic context, estimating an average incidence of neonatal sepsis of 2202 cases per 100,000 live births (95% CI 1099–4360). According to the authors, this result may be hampered by the heterogeneity of the studies included in the meta-analysis.^16^ Comparison of the data obtained in the present study with other reviews should be carried out with extreme caution, as the hospitalizations for neonatal sepsis analyzed here were selected based on the main diagnoses considered by the hospital. Thus, this study shows the incidence of hospitalizations in which neonatal sepsis was considered one of the main diagnoses by the professional who reported the patient to SUS. In this context, the fact that clinical sepsis has been reported does not mean that an etiological agent has not been isolated, but rather that the notification of hospitalization for sepsis was not accompanied by information about its etiological agent. In addition, it is possible that sepsis has been reported, but this diagnosis was excluded after the report. It is also possible that sepsis was not chosen as the main diagnosis in neonates with sepsis. Besides the problems cited above, the prevalence of sepsis found in the study used all Brazilian live births by year, not restricted to live births from public hospitals. The database of Hospital Admission Authorizations used to collect the study information is not linked to the livebirths database and it was not possible to determine whether the newborns’ place of birth was public or private. Such issues restrict any discussion of the incidence of neonatal sepsis with the data presented here and are one of the important limitations arising from the use of secondary data.

Despite this limitation, the comparison of the incidence of hospitalization for neonatal sepsis according to the region of Brazil shows significant variation. Possible explanations for this variation would be the fact that the clinical presentation of neonatal sepsis is non-specific, with signs and symptoms that can simulate other clinical conditions and, therefore, different definitions of the presence of sepsis have been used in different regions from the country. The possibility of hospitalizations of newborns due to sepsis in these regions being underreported is reinforced when observing the distribution of doctors in the national territory. According to the document “Medical Demography in Brazil 2020”, published by the Federal Council of Medicine and the University of São Paulo, the country has never registered so many medical professionals, but the population does not benefit equally from them.^17^

Regarding the frequency of the various etiological agents, the very high presence of unspecified agents may be due to some factors, such as the probable underreporting and using generic infection codes. The hypothesis of low performance of support laboratories in the national hospitals linked to the Unified Health System can also be raised. For all the alternatives mentioned above, there is a need for medical education and training in relation to the diagnosis of sepsis and awareness that accurate reporting may help managers to develop projects to improve the quality of neonatal care.

The portrait of neonatal sepsis lethality according to the region of the country cannot be interpreted in a simplified way, as it results from complex factors derived from socioeconomic inequalities, inequalities in access to the health system and in the quality of perinatal care that interact in multiple ways. It can be observed a tendency of reduction in the lethality rates in all Brazilian regions over the years of the study, which may be associated with better prevention of early sepsis by the universal screening for Streptococcus agalactiae among pregnant women.^18^ In addition, several initiatives may have acted together to decrease the sepsis lethality over the study years, such as the awareness campaign launched by the World Health Organization in 2009, entitled “Save lives: hygiene your hands”,^19^ the national action plan to improve the quality of neonatal care, for example, the “Qualineo” strategy,^20^ and the debriefing on data gathered from national studies such as “Born in Brazil: National survey on labor and birth”.^21^ Such initiatives allowed the construction of strategies with an impact on the incidence of early and late neonatal sepsis and mortality.

In the present study, the mean cost of hospitalization for newborns with sepsis was US$ 3345.59 per patient. A North American multicenter study published in 2001, with 192,980 patients with documented infection and with acute organ dysfunction that included children and adults, showed a mean cost per patient of US$ 22,100.00, with higher mean costs for hospitalizations of children under one year of age (US$ 54,300.00).^22^ On the other hand, the value found in the present study is higher than the average cost per patient indicated by Neira et al.,^6^ who studied the epidemiology and costs of sepsis in Brazil between 2006 and 2015: the mean value per patient with sepsis admitted to the ICU was US$ 1,798.00, including all age groups, with no specific data for the neonatal group. The costs presented by Neira et al., similarly to ours, refer to what the government reimbursed to hospitals for hospitalization for sepsis, according to the reported diagnosis. Arefian et al., in a meta-analysis that encompassed 37 studies with no predetermined age limit, showed an average hospital cost for sepsis ranging from US$ 20,745.00 to US$ 40,835.00.^8^ A possible explanation for the differences in the average cost of hospitalizations in our study in relation to those found in other studies would be the methodology used to calculate these costs. The different calculation methods, the systematic and the detailing of the processes involved in the hospitalization could explain such variability. Also, the value reported by our study is the amount paid by the SUS for the hospitalization, not necessarily revealing the real expense related to it.

The database used in this study presents the values transferred by the SUS to the hospitals. According to the website of the National Health Fund (FNS), the transfer is made through the presentation of an invoice, based on a chart from the Ministry of Health that specifies the value of each care procedure.^23^ It is important to distinguish that the amount transferred by the SUS to the hospital that cares for a newborn with sepsis is different from what was actually spent on the hospitalization of the patient. Therefore, the values presented and discussed in this study reflect how much the government pays for hospitalizations for neonatal sepsis and not necessarily how much it was spent during the care provided to the newborn hospitalized for neonatal sepsis. It is essential that the values reimbursed by the system reflect the real costs of procedures for the very survival of the Brazilian Unified Health System.^24^

The evaluation of costs related to neonatal sepsis in the country during an 11-year period reported in this study shows the economic impact of morbidity that may be avoided by improving the quality of neonatal care. The limitations of the study, highlighted in the discussion, suggest the need for better data collection, both within the scope of hospital reports to the system and in terms of better knowledge of expenditures.

## Authors' contributions

Study design: Mariana FCW de Abreu, Joice FM Ogata, Marcelo CM Fonseca, Ruth Guinsburg.

Data collection: Mariana FCW de Abreu, Dayan Sansone, Marcelo CM Fonseca.

Data analysis: Mariana FCW de Abreu, Joice FM Ogata, Marcelo CM Fonseca, Ruth Guinsburg.

Manuscript writing: Mariana FCW de Abreu, Ruth Guinsburg.

Manuscript revision: Mariana FCW de Abreu, Joice FM Ogata, Dayan Sansone, Marcelo CM Fonseca, Ruth Guinsburg.

Study supervision: Marcelo CM Fonseca, Ruth Guinsburg.

## Funding source

Nothing to declare.

## Conflicts of interest

The authors declare no conflicts of interest.
